# Long-term outcome of a treat-to-target strategy in late-onset rheumatoid arthritis with chronic lung disease: 5-year results of a prospective observational study

**DOI:** 10.1186/s13075-025-03491-1

**Published:** 2025-02-03

**Authors:** Manami Nomura, Takahiko Sugihara, Hiroyuki Baba, Tadashi Hosoya, Mari Kamiya, Tatsuro Ishizaki, Takumi Matsumoto, Kanae Kubo, Fumio Hirano, Masayo Kojima, Nobuyuki Miyasaka, Shinsuke Yasuda, Masayoshi Harigai

**Affiliations:** 1 Department of Rheumatology, Graduate School of Medical and Dental Sciences, Institute of Science Tokyo, Tokyo, Japan; 2 Department of Medicine and Rheumatology, Tokyo Metropolitan Institute for Geriatrics and Gerontology, Tokyo, Japan; 3 Human Care Research Team, Tokyo Metropolitan Institute for Geriatrics and Gerontology, Tokyo, Japan; 4 Present Address: Division of Rheumatology, Department of Internal Medicine, Toho University School of Medicine, 6-11-1 Omori-Nishi, Ota-ku, Tokyo, 143-8541 Japan; 5 Department of Frailty Research, Center for Gerontology and Social Science, National Center for Geriatrics and Gerontology, Aichi, Japan; 6 Nagoya City University Graduate School of Medical Sciences, Aichi, Japan; 7 Department of Rheumatology, Sanno Hospital, Tokyo, Japan; 8 Department of Rheumatology, International University of Health and Welfare, Narita, Japan

**Keywords:** Late-onset rheumatoid arthritis, Treat-to-target, Interstitial lung disease, Chronic lung disease, Simplified disease activity index remission, Health assessment questionnaire disability index, Serious adverse events, Methotrexate, Biological disease-modifying antirheumatic drugs

## Abstract

**Background:**

Controlling disease activity and improving physical function would be more difficult in patients with late-onset rheumatoid arthritis (LORA) who have chronic lung disease (CLD) at baseline. Our aim was to evaluate 5-year outcomes of following a treat-to-target (T2T) strategy targeting low disease activity (LDA) in LORA with CLD.

**Methods:**

Data from 197 methotrexate (MTX)-naïve LORA patients (mean age 74.4 years) from a prospective, monocentric registry were analyzed. Patients were treated with MTX if they had one or more poor prognostic features. If they had interstitial lung disease (ILD), tacrolimus could be administered instead of MTX at the discretion of the attending physician. If patients exhibited no response according to the European League Against Rheumatism criteria at week 12 or had not achieved LDA by week 24, biological disease-modifying antirheumatic drugs (bDMARDs) were started targeting LDA. The primary outcomes were the 5-year simplified disease activity index (SDAI) remission and Health Assessment Questionnaire Disability Index (HAQ-DI) ≤ 0.5 by non-responder imputation analysis. Secondary outcomes were serious adverse events (SAEs).

**Results:**

Of the 197 LORA patients, 47 had CLD at baseline. The proportion of patients using MTX at baseline was significantly lower in those with than without CLD. Tacrolimus was initiated in 25.5% of the CLD group. The proportion of patients on bDMARDs was higher in those with CLD at year 5. Achievement of SDAI remission at year 5 was 29.8% in patients with CLD and 44.0% in those without CLD (*p* = 0.555). Achievement of HAQ-DI ≤ 0.5 at year 5 was 36.2% and 45.3% in patients with and without CLD, respectively (*p* = 0.939). Non-adherence to T2T due to comorbidities or adverse events was observed in 34.0% and 18.7% of the patients with and without CLD, respectively (*p* = 0.027). Infections requiring hospitalization, deterioration of extra-articular manifestations and fractures were more frequently reported as SAEs in patients with CLD, and multivariable analysis showed that patients with CLD had a higher risk of developing these SAEs (adjusted hazard ratio:2.53, 95% CI 1.60–4.00, *p* < 0.001).

**Conclusion:**

For LORA patients with CLD, the T2T strategy is effective, but comorbidities and SAEs make the implementation of the T2T more difficult.

**Supplementary Information:**

The online version contains supplementary material available at 10.1186/s13075-025-03491-1.

## Backgrounds

A treat-to-target (T2T) strategy is the gold standard treatment protocol for patients with rheumatoid arthritis (RA), and achieving remission is the initial treatment goal for methotrexate (MTX)-naïve patients. Older patients with RA tend to have more comorbidities and be at a higher risk for serious infections and drug-related adverse events (AEs) than younger patients [1–3]. Older age and chronic lung disease (CLD) are known risk factors for MTX-induced lung injury [4, 5], leading to less frequent use of MTX in patients with CLD [6]. Of these, older RA patients with CLD are at potential risk of becoming difficult-to-treat cases due to limited drug tolerance [7, 8]. Additionally, patients with interstitial lung disease (ILD) have significantly increased mortality relative to patients without ILD [9, 10].

We conducted a prospective cohort (the Choju registry of RA treated with non-biologic disease-modifying anti-rheumatic drugs and biologics in elderly patients in Japan (CRANE) cohort) study to evaluate the effectiveness and safety of the T2T strategy targeting low disease activity (LDA) in patients with late-onset rheumatoid arthritis (LORA) [11]. Our previous report from CRANE indicated that high disease activity, anti-citrullinated protein antibody (ACPA) positivity, bony erosion at baseline, no treatment response at week 12, and non-achievement of LDA at week 24 were all predictors of one-year clinically relevant radiographic progression (CRRP) [11]. The proportion of remission according to the simplified disease activity index (SDAI) and Health Assessment Questionnaire Disability Index (HAQ-DI) ≤ 0.5 was significantly higher in patients adhering to T2T than those not adhering to T2T at year 3 [12].

We hypothesized that controlling disease activity and improving physical function would be more difficult in patients with LORA who have CLD at baseline. In the present study, we evaluated the 5-year effectiveness and safety of a T2T strategy in patients with LORA, specifically focusing on those with CLD at baseline.

## Methods

### Data source

CRANE is a prospective monocentric cohort from the Tokyo Metropolitan Geriatric Hospital, enrolled between 2008 and 2015. Patients were included in the study if they met the 1987 revised American College of Rheumatology (ACR) classification criteria for RA, were diagnosed at ≥ 60 years of age, were MTX-naïve, and had a moderate-to-high disease activity with 28-joint disease activity score using erythrocyte sedimentation rate (DAS28-ESR) of ≥ 3.2. The patients received treatment targeting LDA (i.e., SDAI of < 11.0 or DAS28-ESR of < 3.2) based on a T2T strategy, and the effectiveness and safety were prospectively evaluated over 5 years from baseline. Data were extracted at weeks 0, 12, 24, 36, 52, 76, 104, 128, 156, 180, 208, 232, and 260. The Ethics Committee of Tokyo Metropolitan Geriatric Hospital approved the protocol of this study (240117, 467) and all patients provided written informed consent.

### Treatment protocol

The treatment protocol was described in a previous study of 1-year and 3-year outcomes in the CRANE cohort (Supplementary Figure S1) [11, 12]. Patients were treated with MTX if they had one or more poor prognostic features, such as high disease activity, positive ACPA, low physical function, or bony erosion by radiography at diagnosis. If the patients have no poor prognostic factors, Starting other csDMARDs such as salazosulfapyridine (SASP) instead of MTX was acceptable at the discretion of the attending physician (Supplementary Figure S1). If they had ILD, tacrolimus could be administered at the discretion of the attending physician. Low-dose glucocorticoids (GCs) were started also at the discretion of the attending, without any pre-determined dose reduction. If patients exhibited no response according to the European League Against Rheumatism (EULAR) criteria at week 12 or had not achieved LDA by week 24, tumor necrosis factor inhibitors (TNFi) were started following the 2008 ACR recommendations and the 2008 Japan College of Rheumatology (JCR) guidelines [13, 14]. If the first TNFi was ineffective, it was changed to a different TNFi, tocilizumab, or abatacept. Non-implementation of the T2T strategy was defined as no treatment intensification despite moderate or high disease activity using SDAI at 24 weeks, 36 weeks, 52 weeks, and every 24 weeks after that up to 256 weeks of observation. The reasons for non-adherence were dichotomized as either the patient’s decision or the presence of comorbidities / AEs.

### Outcomes

The primary outcomes were SDAI remission and HAQ-DI ≤ 0.5 by the non-responder imputation (NRI) method in LORA patients with and without CLD at year 5. Secondary outcomes were SDAI LDA at years 2 and 5, SDAI remission and HAQ-DI ≤ 0.5 at year 2 and the nonadherence proportion of T2T. SAEs of special interest were infections requiring hospitalization, deterioration of extra-articular manifestations including exacerbation of ILD, fractures, cardiovascular disease (CVD) requiring hospitalization, and malignancies. When a patient reported more than one event for each SAE of special interest, all of them were counted. The patients received treatment targeting LDA even after the occurrence of SAEs according to the protocol when considered clinically appropriate by an attending physician. The observation was censored in each patient at year 5, increasing GCs for extra-articular manifestations such as ILD, death, or lost-to-follow-up, whichever came first. After increasing the dose of GCs for worsening extra-articular manifestations, a subsequent clinical course was followed outside of the observation period up to 5 years from the start of T2T.

### Definition and identification of CLD

CLD at baseline encompassed ILD, emphysema, or airway disease. The airway disease included chronic bronchitis, bronchiolitis, or bronchiectasis. The CLD was diagnosed as either of the following: (1) ILD, emphysema, or airway disease diagnosed by a pulmonologist prior to entry into the cohort, (2) High-resolution computed tomography (HRCT) was conducted for patients with respiratory symptoms or suspected abnormal findings on chest X-P, and ILD, emphysema, and airway disease were diagnosed by both a radiologist and a rheumatologist.

### Statistics

Student‘s *t*-test and the Mann-Whitney test were used to compare continuous variables depending on their distribution, and the chi-square test and Fisher’s exact test were used for categorical variables. The cumulative rates and median time to the first event leading to treatment discontinuation were analyzed using the Kaplan-Meier method with the log-rank test. We examined how the presence of CLD influenced the 5-year SAE incidence rate by using the univariable and multivariable Cox proportional hazards model, of which results were expressed as an adjusted hazards ratio (HR) with a 95% confidence interval (CI). Age, SDAI, HAQ-DI, GCs use, history of smoking and comorbidities (estimated creatinine clearance (Ccr) < 60 ml/min determined by the Cockcroft-Gault formula, diabetes, osteoporosis, history of infections requiring hospitalization, and history of malignancy) at baseline were selected as confounding factors of multivariable Cox proportional hazards model. All analytical procedures were performed using IBM SPSS version 27 (IBM Corp., Armonk, NY, USA). All reported *P*-values are two-tailed, and the level of significance was set at *P* < 0.05.

## Results

### Baseline characteristics of LORA patients with and without CLD

Of the 197 patients with LORA, 47 had CLD at baseline (ILD, *n* = 31; airway disease, *n* = 12; emphysema, *n* = 10; others, *n* = 2). There were no significant differences between patients with (CLD group) or without CLD (non-CLD group) in terms of age, sex, disease duration, SDAI, DAS28, HAQ-DI, and bony erosion (van der Heijde-modified total Sharp score [mTSS] ≥ 2) at baseline (Table 1). Patients with CLD had significantly higher prevalences of ACPA positivity and history of infections requiring hospitalization. Smoking history was numerically more common in patients with CLD.

**Table 1:** Baseline characteristics and T2T implementation of LORA patients with and without CLD

	With CLD *n* = 47	Without CLD *n* = 150	*p*-value
Age, years, mean (S.D.)	74.1(6.1)	74.5(7.0)	0.737
Sex, female, %	68.1	72.7	0.543
Symptom duration, years, median (IQR)	0.6(0.5–2.0)	0.5(0.25–1.35)	0.117
Body weight, kg, mean (S.D.)	50.1(9.9)	53.4(10.1)	0.054
ACPA-positive, %	85.1	62.7	0.004
SDAI at week 0, mean (S.D.)	37.8(15.8)	35.6(16.6)	0.438
DAS28-ESR, mean (S.D.)	6.30 (1.17)	6.03 (1.19)	0.194
Erosion score $\:\ge\:$2, %	63.0	49.0	0.096
HAQ-DI at week 0, median (IQR)	1.0 (0.38–1.88)	0.875 (0.47–1.63)	0.385
History of smoking, %	51.1	35.3	0.054
History of infections requiring hospitalization, %	17.0	6.0	0.025
Ccr < 60 ml/min at baseline, %	48.9	40.3	0.294
CVD at baseline, %	27.7	11.3	0.007
History of malignancy, %	12.8	10.0	0.592
Nonadherence to T2T, %	22 (46.8%)	53 (35.3%)	0.157
Failed to adhere on one occasion	9 (19.1%)	25 (16.7%)	
Failed to adhere on twice	5 (10.6%)	14 (9.3%)	
Failed to adhere on three times	3 (6.4%)	8 (5.3%)	
Failed to adhere on four times	2 (4.3%)	2 (1.3%)	
Failed to adhere on five times or more	3 (6.4%)	4 (2.7%)	
Non-adherence to the T2T due to comorbidities or AEs	16 (34.0%)	28 (18.7%)	0.027
Non-adherence to the T2T patient´s own decision	10 (21.3%)	32 (21.3%)	0.993
Discontinuation of observation	24 (51.1%)	51 (34.0%)	0.036
Follow-up time, week, mean (S.D.)	197.6 (82.0)	217.8 (72.8)	0.110

LORA: late-onset rheumatoid arthritis, CLD: chronic lung disease, S.D.: standard deviation, ACPA: anti-citrullinated protein antibody, SDAI: simplified disease activity index, DAS: disease activity score, HAQ-DI: Health Assessment Questionnaire Disability Index, IQR: Interquartile Range, Ccr; creatinine clearance, CVD: cardiovascular disease, T2T: treat-to-target, AEs: adverse events

^*^Statistically significant with *p* < 0.05

### Treatment during the 5-year observation period

MTX was started in 79.7% of the 197 patients at baseline, and prescribed to 76.9% of the 173 patients remaining in the study at year 2 and 68% of the 122 remaining at year 5. TNFi, IL-6 inhibitors, and abatacept were used in 31.8%, 1.7%, and 1.7% of the 173 patients at year 2, and 20.5%, 5.7%, and 3.3% of the 122 patients at year 5, respectively.

**Fig. 1:**
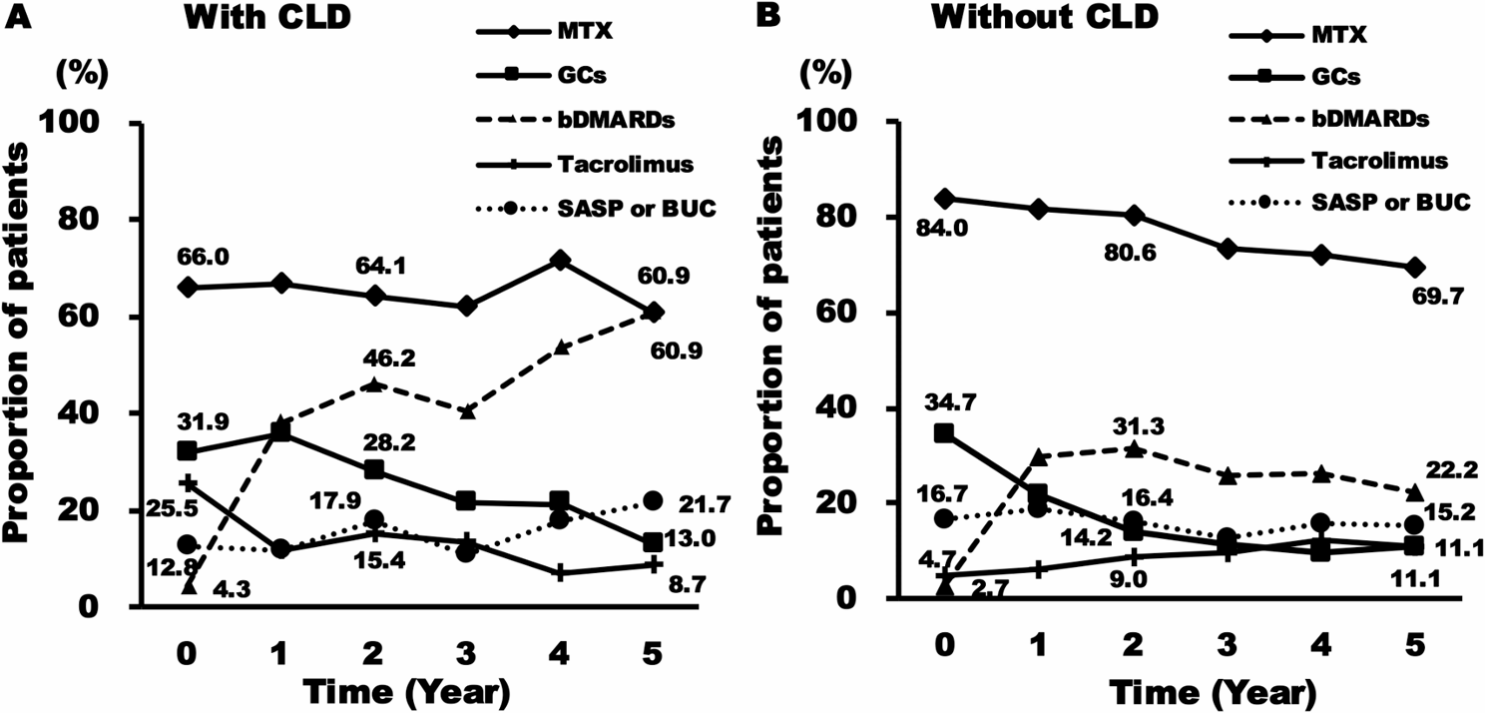
Treatment of LORA patients with and without CLD. Time variation in the proportion of patients with and without CLD taking MTX, GCs, bDMARDs, Tacrolimus, and SASP or BUC. LORA: late-onset rheumatoid arthritis, CLD: chronic lung disease, MTX: methotrexate, GCs: glucocorticoids, bDMARDs: biological disease-modifying antirheumatic drugs (Infliximab, Etanercept, Adalimumab, Golimumab, Certolizumab, Tocilizumab, Abatacept), SASP: Salazosulfapyridine, BUC: Bucillamine

The proportion of patients initiating MTX at baseline was significantly lower in the CLD group compared to the non-CLD group (*p* = 0.007), and also at year 2 (CLD group, 64.1%; non-CLD group, 80.6%; *p* = 0.032). After 3 years, MTX use in patients without CLD decreased to approximately 70% of the patients remaining in the cohort, and by year 5 there was no significant difference between the two groups. Tacrolimus was initiated in 25.5% of the CLD group, declining to 15.4% at 2 years and 8.7% at 5 years (Fig. 1A). The proportion of patients prescribed with GCs decreased from 31.9% at baseline to 13.0% at year 5 in patients with CLD (Fig. 1A) and from 34.7% at baseline to 11.1% at year 5 in patients without CLD (Fig. 1B). The mean daily dose of GCs in patients with and without CLD was 6.0 (S.D. 1.8) mg and 6.2 (S.D. 2.3) mg of prednisolone equivalent at baseline and 2.3 (S.D.0.6) mg and 3.8 (S.D.2.0) mg at 5 years, respectively. The percentage of patients using biological disease-modifying antirheumatic drugs (bDMARDs) increased from 46.2% at year 2 to 60.9% at year 5 in patients with CLD (Fig. 1A), but decreased from 31.3% at year 2 to 22.2% at year 5 in those without CLD (Fig. 1B). Thus, the proportion of patients on bDMARDs at year 5 was significantly higher in the CLD group compared to non-CLD group (*p* < 0.001).

### Discontinuation of observation

**Fig. 2:**
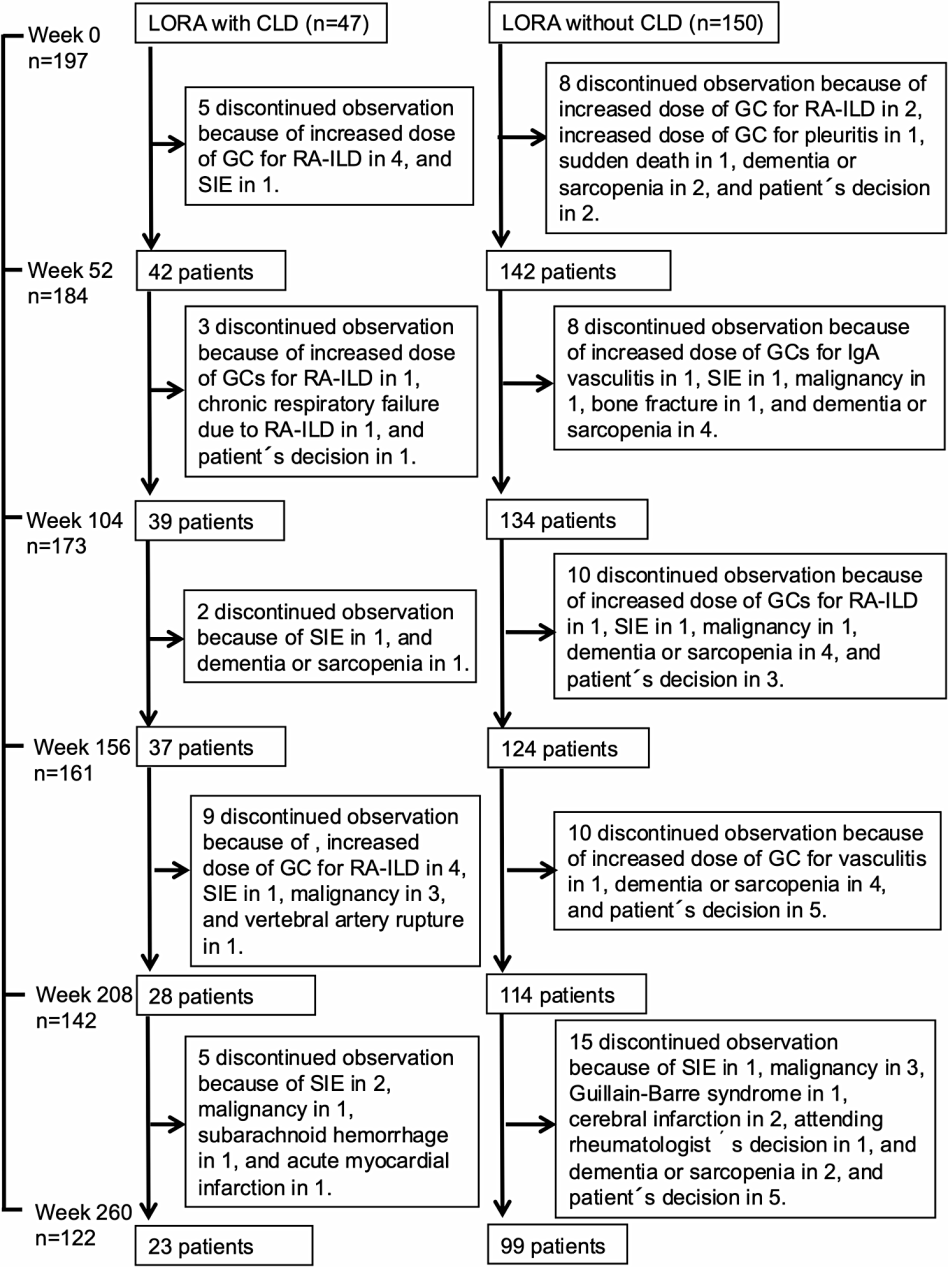
Flow chart of the study. Twenty-three of 44 patients with CLD and 99 of 150 patients without CLD completed observation over five years. The flow chart shows the reasons for the discontinuations of observation in LORA with and without CLD. LORA: late-onset rheumatoid arthritis, CLD: chronic lung disease, GCs: glucocorticoids, ILD: Interstitial lung disease; SIE: serious infectious event

The number of patients with events leading to discontinuation of observation was 24 (51.1%) of 47 patients with CLD and 51 (34.0%) of 150 without CLD (Table 1; Fig. 2). Increased dose of GCs for RA-ILD accounted for 9 (37.5%) of the 24 patients with CLD and 3 (5.9%) of the 51 patients without CLD. Serious infectious events (SIEs) were reported in 5 (20.8%) of the 24 patients with CLD and 3 (5.9%) of the 51 patients without CLD. Age-related difficulties in physically accessing an outpatient clinic were reported in 1 (4.2%) of the 24 events in patients with CLD and 16 (31.4%) of the 51 events in patients without CLD.

### Long-term outcomes in patients with and without CLD

**Fig. 3:**
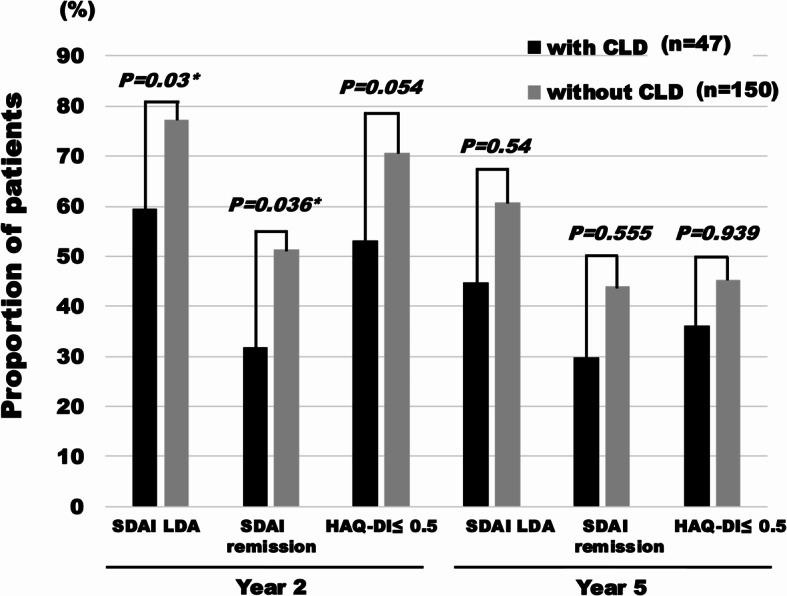
Long-term outcomes in patients with and without CLD. Non-responder imputation approaches were applied to estimate the proportion of achievement of treatment outcomes. CLD: chronic lung disease, HAQ-DI: Health Assessment Questionnaire Disability Index, LDA: low disease activity; SDAI: simplified disease activity index. ^*^Statistically significant with *p* < 0.05

SDAI remission by NRI analysis of LORA patients with and without CLD was achieved by 31.9% and 51.3%, respectively, at year 2 (*p* = 0.036, Fig. 3). At year 5, these values were 29.8% and 44.0%, respectively, which were no longer significantly different (*p* = 0.555, Fig. 3). In the last observation carried forward (LOCF) analysis, the proportion of SDAI remission at year 5 was 55.3% in patients with CLD and 57.3% in patients without. According to NRI, HAQ-DI ≤ 0.5 was achieved by 53.2% of the patients with CLD at year 2 and by 36.2% at year 5, while these values were 70.7% and 45.3% of patients without CLD, respectively (Fig. 3). These differences between the two groups were not statistically significant, but the rates were numerically higher in LORA without CLD.

### Adherence to the T2T strategy

The nonadherence proportion of T2T was 46.8% of the 47 patients with CLD and 35.3% of the 150 without CLD, and these were not significantly different (Table 1). Treatment could not be intensified due to comorbidities or AEs in 34.0% of the patients with CLD and 18.7% of the patients without CLD, and these were significantly different (*p* = 0.027). Non-adherence due to the patient´s own decision was similarly reported in two groups.

### Treatment outcomes in patients with and without CLD who adhered to the T2T

At year 2, patients with CLD who adhered to the T2T throughout the observational periods achieved SDAI remission and HAQ-DI ≤ 0.5 at 44.0% and 68.0% by NRI analysis, respectively, and these rates were numerically higher than those of the patients with non-adherence at least once on their visits (SDAI remission: 18.2%, HAQ-DI ≤ 0.5: 36.4%) (Supplementary Table S1). A similar trend was observed after 5 years (T2T adherence group: SDAI remission: 32.0% by NRI and 64.0% by LOCF, non-adherence group: SDAI remission: 27.3% by NRI and 45.5% by LOCF) (Supplementary Table S1). In patients without CLD, adherence to T2T resulted in significantly higher remission rates at 2 years in the NRI analysis and 2 and 5 years in the LOCF. The remission rate at 5 years by NRI was numerically higher in patients without CLD who adhered to T2T (Supplementary Table S1).

### Exacerbation of pre-existing ILD and occurrence of new-onset ILD during the 5-year observation period

Incident rates per 100 person-years were 1.84 for ILD or pleuritis. Of the 47 RA patients with CLD at baseline including 31 patients with ILD (ILD group), 10 experienced worsening of pre-existing ILD during the 5-year observation period. The mean time to the event was 97.3 weeks (standard deviation (S.D.) 76.3, minimum 4, maximum 192). Of the 18 patients who started MTX in the ILD group, 4 (22.2%) patients had worsening of ILD. On the other hand, 5 (50.0%) of the 10 patients starting tacrolimus in the ILD group showed worsening of ILD. As the cumulative disease activity before the exacerbation of ILD, the average SDAI for the period from the start of treatment to the worsening of ILD (SDAI _0−ILD_) was calculated. The mean SDAI _0−ILD_ of the 10 patients with worsening pre-existing ILD was 29.3 (S.D. 11.9). On the other hand, the mean SDAI during the observation period was 13.0 (S.D. 4.9) for the ILD group without worsening ILD. These were statistically significantly different (*p* = 0.002).

Of 150 RA patients without CLD at baseline, two newly developed ILD other than OP, two developed OP and one developed pleuritis. The mean time to the event was 60.8 weeks (S.D. 46.2, minimum 12, maximum 116).

Four patients in the CLD group and one in the non-CLD group were transferred to a long-term care hospital without being able to return home due to activities of daily living (ADL) decline after high-dose GCs treatment for ILD exacerbation (Table 2). Death after pre-existing ILD exacerbation was reported in three patients. One patient died of severe infection and stroke nine months after high-dose GCs and cyclophosphamide treatment for ILD exacerbation. One patient died of recurrent ILD 15 months after starting treatment with increased GCs dose and abatacept for worsening of ILD. One patient had persistent high disease activity and died of acute exacerbation of ILD 8 months after starting T2T.

**Table 2:** Exacerbation of RA-associated lung diseases during the 5-year observation period

	with CLD *n* = 47	without CLD *n* = 150
Exacerbation of RA-associated lung diseases, n	10	5
Exacerbation of prevalent ILD, n	10	0
New-onset of ILD other than OP, n	0	2
New-onset of OP, n	0	2
New-onset of pleuritis, n	0	1
Event occurrence, week, mean (S.D.)	97.3 (76.3)	60.8 (46.2)
Increased dose of GCs, n	9	4
Cessation of hospital visits due to decline in ADL after worsening of ILD*, n	4^†^	1^†^
Death after ILD exacerbation, n	3^†^	0^†^

* In Japan, after treating acute medical conditions such as ILD exacerbations, patients are transferred to long-term care hospitals when they cannot return home due to a decline in ADL. Information after transfer was not available in this study

^†^ Clinical course after exacerbation of RA-associated lung diseases was followed outside of the observation period up to 5 years from the start of T2T

RA: rheumatoid arthritis, ILD: Interstitial lung disease, OP: organizing pneumonia, S.D.: standard deviation, GCs: glucocorticoids, SIE: Serious infectious event,

ADL: activities of daily living, T2T: treat-to-target

### SAEs during the 5-year observation period

SAEs of special interest were noted in 82 patients during the 5-year observation period. The total observation time for all 197 patients was 814 patient-years (PY) at year 5. Incident rates per 100 person-years were 4.05 for infections requiring hospitalization, 1.47 for CVD, 2.09 for malignancy, and 2.21 for bone fractures. Malignancies were reported in 5 (3 had recurrence) of 21 patients (23.8%) with a history of malignancy at baseline and *de novo* in 12 of the 176 patients without such a history (6.8%). Three of these 17 developed lung cancers, 6 prostate cancers, 5 gastrointestinal cancers, and 3 malignant lymphomas. MTX-associated lymphoma was diagnosed in one of these 3 patients.

Incident rates per 100 person-years were 18.7 and 7.59 for SAEs of special interest, 8.79 and 2.69 for infections requiring hospitalization, 6.04 and 1.42 for worsening extra-articular lesions, 3.85 and 1.58 for malignancies, and 4.40 and 1.58 for fractures, in patients with and without CLD, respectively. Lung cancer was reported in 2 of 47 patients with CLD and 1 of 150 patients without (supplementary Table S2).

**Fig. 4:**
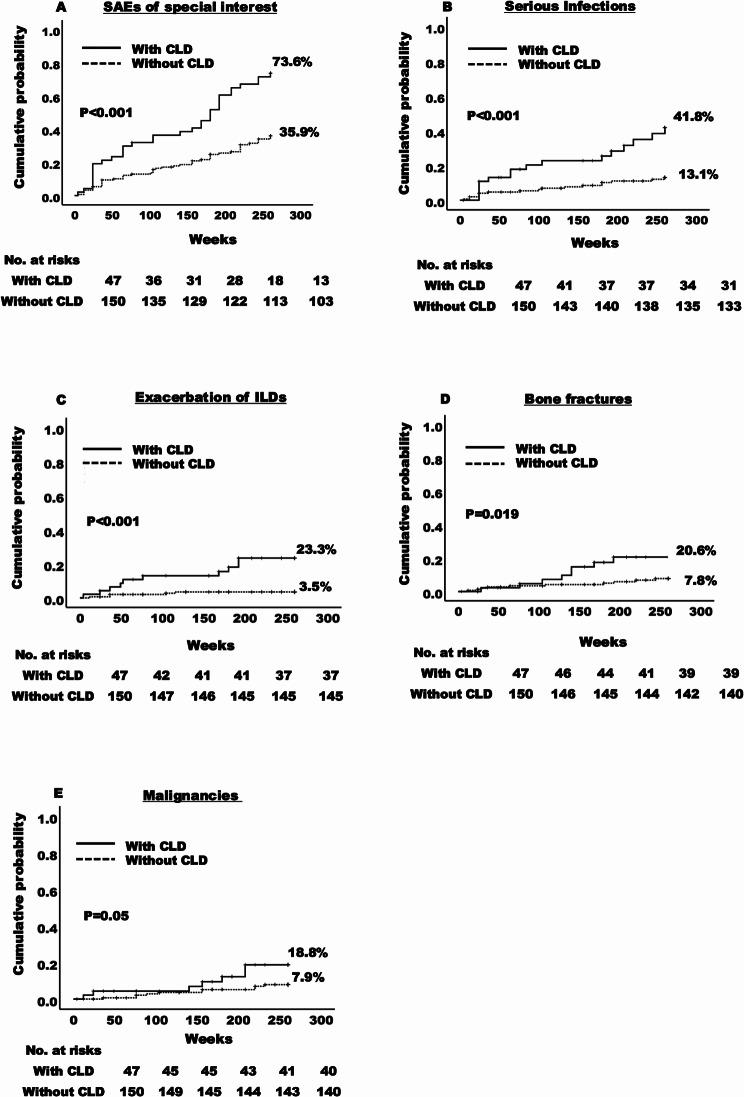
The probability of SAEs of special interest in LORA patients with and without CLD. SAEs of interest were collected, including serious infection, deterioration of ILDs, bone fractures, CVD, and malignancy. The time to the occurrence of SAEs of special interest (*P* < 0.001) (**A**), serious infections (*P* < 0.001) (**B**), deterioration of ILDs (*P* < 0.001) (**C**), malignancies (*P* = 0.05) (**D**), and bone fractures (*P* = 0.019) (**E**) for with and without CLD patients were analyzed using the Kaplan-Meier method. SAEs: serious adverse events, LORA: late-onset rheumatoid arthritis, CLD: chronic lung disease, ILD: Interstitial lung disease, CVD: Cardiovascular diseases. ^*^Statistically significant with *p* < 0.05

A significantly shorter time to the occurrence of SAEs of special interest was recorded for patients with CLD compared to those without CLD (*p* < 0.001, log-rank test). Kaplan-Meier analysis estimated that the cumulative probability of SAEs of special interest was 73.6% for patients with CLD versus 35.9% for those without (Fig. 4A). Multivariable analysis showed that patients with CLD had a higher risk of SAEs of special interest (adjusted HR 2.53, 95% CI 1.60–4.00) versus those without CLD (supplementary Table S3) after adjusting for covariates. Among SAEs of special interest, times to infections requiring hospitalization (Fig. 4B), deterioration of extra-articular manifestations (mainly deterioration of ILD) (Fig. 4C), and fractures (Fig. 4D) were significantly shorter in the CLD group compared to the non-CLD group. Time to onset of malignancies also tended to be shorter (Fig. 4E). During the observation period, six patients deceased, one each of an infection, malignant lymphoma, and subarachnoid hemorrhage in the CLD group, and one each of heart failure, pancreatic cancer, and sudden death in the non-CLD group. As described previously, three patients died of ILD exacerbation outside of the observation period.

## Discussion

We analyzed the long-term outcomes of a T2T strategy targeting LDA in LORA patients with and without CLD at baseline. One important novel finding is that the proportions of patients achieving SDAI remission at year 5, as estimated by NRI, were similar between the two groups, indicating that a T2T strategy targeting LDA is equally effective in the long term for LORA patients either with or without CLD at the start of therapy. However, the proportion of patients non-adherent to T2T due to comorbidities or AEs in the CLD group was significantly higher than that of the non-CLD group, and infections requiring hospitalization, deterioration of extra-articular manifestations, and fractures were more frequently reported in patients with CLD.

Previous reports from the CRANE cohort have documented that adherence to the T2T strategy targeting LDA led to better 3-year outcomes for LORA in terms of controlling disease activity and preventing radiologic progression [12]. The present study found proportions of non-adherence of T2T due to the patient´s own decision during 5 years were similar between patients with and without CLD, and patients with CLD received more bDMARDs than the patients without CLD. The difference in SDAI remission at year 5 between the two groups was not significant. The proportions of SDAI remission at year 5 estimated by LOCF in the CLD and non-CLD group in the present study were almost similar to those of SDAI remission in the long term in previous T2T cohort studies [15–17]. We also confirmed that patients without CLD who adhered to T2T over 5 years had better outcomes. In addition, patients with CLD who were able to adhere to T2T throughout the observation period also had numerically better outcomes in terms of SDAI remission, SDAI LDA, and physical function, compared to those with non-adherence to T2T. These suggest that the T2T strategy, including the long-term use of bDMARDs, may be beneficial in LORA with CLD as well as those without CLD. Since no randomization was performed with and without T2T and the backgrounds of the two groups are different, further research is needed to conclude whether T2T is effective for LORA patients with CLD.

A previous study showed that older age at RA onset [9, 18], ACPA positivity, and smoking history were associated with ILD in patients with RA [19]. A recent Japanese cohort study showed that the time to achieve remission was significantly longer in RA patients with ILD than those without ILD [20]. Previous cohort studies suggested that age at ILD diagnosis [21] and RA disease activity [22] were associated with mortality in RA with ILD, and the persistence of moderate or high disease activity is associated with the newly developed ILD [23]. These suggested that early improvement of disease activity might prevent ILD from developing or worsening in patients with RA. In the CRANE cohort, the LORA with CLD had a lower proportion of remission at 2 years of treatment, and the exacerbation of existing ILD occurred within an average of 2 years from the start of treatment, while LORA without ILD at baseline rarely developed ILD. Cumulative disease activity was higher in patients with worsening preexisting ILD than in those without worsening preexisting ILD. These suggested that early improvement of disease activity might be important for LORA with CLD. However, the number of patients in this study was small, and patients with exacerbations of ILD may have poor disease activity due to treatment limitations and other factors, so further investigation is warranted to determine whether disease activity control is associated with exacerbations of ILD in patients with LORA.

CLD was associated with the development of serious infections in patients with RA during treatment [24, 25], as also shown by post-marketing studies of bDMARDs in Japan [26–28] and in large registries in Europe [29–31] and the US [32, 33]. The present study confirmed the increased risk of serious infections, deterioration of ILD and fractures in LORA with CLD compared to LORA without CLD, and the patients with CLD had a higher proportion of non-adherence of T2T due to comorbidities or AEs than patients without CLD. A recent Japanese cohort study also showed that hospitalized infection, major adverse cardiac events, and lung cancer were more common in RA with ILD than those without ILD [20]. The incidence of serious infection during five years in LORA with CLD in the CRANE cohort was higher than those in younger populations of RA with ILD in other RA cohorts [20, 34]. RA with ILD has been reported to have a higher mortality rate than RA without ILD [9, 20, 35, 36]. The major causes of death in RA with ILD were acute exacerbation of RA-associated ILD, lung cancer, and respiratory infection [10, 36]. Older patients with RA and ILD had higher mortality from respiratory disease and cancer than older patients with RA but without ILD [37]. In the CRANE cohort, ILD worsened in 21.3% of patients with CLD, and 70% of the worsening cases resulted in death or transfer to a long-term care facility (Table 2). These results indicate that older patients with CLD have more difficulty implementing T2T than patients without CLD, and ILD exacerbations, infections, and malignancies should be carefully monitored over time while implementing T2T in patients with LORA.

The present study has several limitations. First, the number of enrolled patients was small and the evaluation was performed at a single center. LORA with CLD tended to have numerically worse outcomes in the present study. When examined in a multicenter study with a larger sample size, patients with CLD may have statistically significantly worse outcomes. Further study is required. Second, this study was not designed to evaluate LORA with CLD, and HRCT or respiratory function test was not performed in all patients, so mild lung lesions may not have been identified at baseline. In addition, the incidence of ILD exacerbations may have been underestimated because the definition of ILD exacerbation was not specified in the scheduled respiratory function tests or the HRCT. Third, MTX is not usually administered based on the Japanese guide for MTX use [38] when ILD is extensive or when lung function is impaired. In this study, since TAC was used instead of MTX at the attending physician’s discretion, the patients who started tacrolimus might have an active ILD and then an indication bias for ILD exacerbation. Fourth, discontinuation of observation due to age-related difficulties in physically accessing an outpatient clinic may have influenced the outcomes of patients without CLD. Fifth, we categorized reasons for not implementing T2T into patient’s decision or comorbidities / AEs, but we did not collect breakdowns of these two categories.

## Conclusion

The T2T strategy targeting LDA improved disease activity and physical function in the long term for LORA patients with CLD at baseline as well as for LORA patients without CLD. However, serious infections, deterioration of ILD, and fractures were observed more frequently in LORA with CLD. More evidence is required regarding the management of ILD and SAEs when implementing T2T for LORA patients with CLD.

## Electronic supplementary material

Below is the link to the electronic supplementary material.

Supplementary Material 1

Supplementary Material 2

## Data Availability

No datasets were generated or analysed during the current study.

## Abbreviations

ACPA: Anti-citrullinated protein antibody
ACR: American College of Rheumatology
ADL: Activities of daily living
AEs: Adverse events
bDMARDs: Biological disease-modifying antirheumatic drugs
BUC: Bucillamine
Ccr: Creatinine clearance
CRANE: Choju registry of RA treated with non-biologic DMARDs and biologics in elderly patients in Japan
CVD: Cardiovascular disease
DAS: Disease activity score
ESR: Erythrocyte sedimentation rate
EULAR: European League Against Rheumatism
GCs: Glucocorticoids
HAQ-DI: Health Assessment Questionnaire Disability Index
ILD: Interstitial lung disease
IQR: Interquartile range
JCR: Japan College of Rheumatology
LDA: Low disease activity
LOCF: Last observation carried forward
LORA: Late-onset rheumatoid arthritis
mTSS: Van der Heijde-modified total Sharp score
MTX: Methotrexate
NRI: Non-responder imputation
OP: Organizing pneumonia
RA: Rheumatoid arthritis
SASP: Salazosulfapyridine
S.D.: Standard deviation
SDAI: Simplified disease activity index
SIE: Serious infectious event
TNFi: Tumor necrosis factor inhibitors
T2T: Treat-to-target
