# Supplementary figures and images for: Long-term outcome of a treat-to-target strategy in late-onset rheumatoid arthritis with chronic lung disease: 5-year results of a prospective observational study
Source: Arthritis Res Ther. 2025 Feb 3;27:22. doi: 10.1186/s13075-025-03491-1 (PMC11789366; doi:10.1186/s13075-025-03491-1)

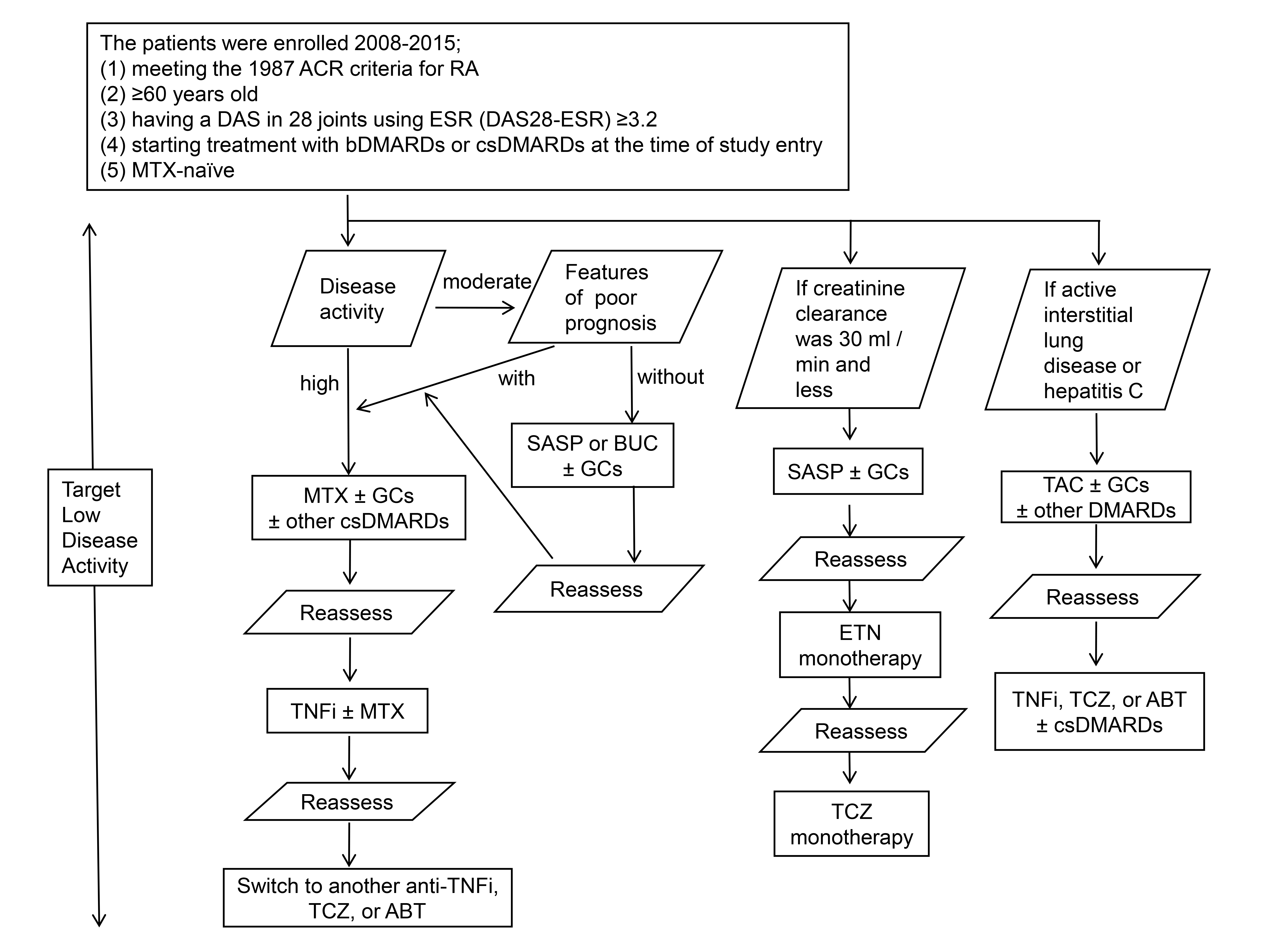

Supplement: Supplementary file 1 — Supplementary Material 1 [file 13075_2025_3491_MOESM1_ESM.tif]
