# Supplementary material for: Long-term outcome of a treat-to-target strategy in late-onset rheumatoid arthritis with chronic lung disease: 5-year results of a prospective observational study
Source: Arthritis Res Ther. 2025 Feb 3;27:22. doi: 10.1186/s13075-025-03491-1 (PMC11789366; doi:10.1186/s13075-025-03491-1)
Supplement: Supplementary file 2 — Supplementary Material 2 [file 13075_2025_3491_MOESM2_ESM.docx]

Supplementary Table S1 Treatment outcomes in patients with and without CLD who adhered to the T2T

NRI

|  |  |  | Adherence | Non-adherence | *p*-value |
| --- | --- | --- | --- | --- | --- |
| 2-year | With CLD | SDAI LDA, % | 72 | 45.5 | 0.158 |
|  |  | SDAI remission, % | 44.0 | 18.2 | 0.092 |
|  |  | HAQ-DI≦0.5, % | 68.0 | 36.4 | 0.051 |
|  | Without CLD | SDAI LDA, % | 86.6 | 60.4 | <0.001* |
|  |  | SDAI remission, % | 66.0 | 24.5 | <0.001* |
|  |  | HAQ-DI≦0.5, % | 75.3 | 62.3 | 0.243 |
| 5-year | With CLD | SDAI LDA, % | 44 | 45.5 | 0.478 |
|  |  | SDAI remission, % | 32.0 | 27.3 | 0.400 |
|  |  | HAQ-DI≦0.5, % | 40.0 | 31.8 | 0.155 |
|  | Without CLD | SDAI LDA, % | 66.0 | 50.9 | 0.672 |
|  |  | SDAI remission, % | 49.5 | 34.0 | 0.303 |
|  |  | HAQ-DI≦0.5, % | 51.5 | 34.0 | 0.369 |

LOCF

|  |  |  | adherence | non-adherence | *p*-value |
| --- | --- | --- | --- | --- | --- |
| 2-year | With CLD | SDAI LDA, % | 76 | 54.5 | 0.075 |
|  |  | SDAI remission, % | 44.0 | 27.3 | 0.193 |
|  |  | HAQ-DI≦0.5, % | 72.0 | 40.9 | 0.019* |
|  | Without CLD | SDAI LDA, % | 93.8 | 66.0 | <0.001* |
|  |  | SDAI remission, % | 68.0 | 26.4 | <0.001* |
|  |  | HAQ-DI≦0.5, % | 78.4 | 67.9 | 0.128 |
| 5-year | With CLD | SDAI LDA, % | 88 | 72.7 | 0.128 |
|  |  | SDAI remission, % | 64.0 | 45.5 | 0.147 |
|  |  | HAQ-DI≦0.5, % | 68.0 | 54.5 | 0.253 |
|  | Without CLD | SDAI LDA, % | 93.8 | 67.9 | <0.001* |
|  |  | SDAI remission, % | 66.0 | 41.5 | 0.004* |
|  |  | HAQ-DI≦0.5, % | 72.2 | 56.6 | 0.053 |

LOCF and NRI approaches were applied to estimate the proportion of achievement of treatment outcomes.

CLD: chronic lung disease, HAQ-DI: **Health Assessment Questionnaire Disability Index**, LDA: low disease activity, LOCF: last observation carried forward, NRI: non-responder imputation, SDAI: simplified disease activity index.

^*^Statistically significant with p <0.05.

Supplementary Table S2 Malignancy during the 5-year observation period in LORA patients with and without CLD.

|  | n=197 | with CLD  n=47 | without CLD n=150 |
| --- | --- | --- | --- |
| lung cancer | 3 | 2 (4.3%) | 1 (0.7%) |
| prostate cancer | 6 | 1 (2.1%) | 5 (3.3%) |
| esophageal cancer | 2 | 1 (2.1%) | 1 (0.7%) |
| stomach cancer | 1 | 1 (2.1%) | 0 (0%) |
| transverse colon cancer | 1 | 1 (2.1%) | 0 (0%) |
| malignant lymphoma | 3 | 1 (2.1%) | 2 (1.3%) |
| pancreas cancer | 1 | 0 (0%) | 1 (0.7%) |

The cumulative rate of lung cancer, prostate cancer, esophageal cancer, stomach cancer, transverse colon cancer, malignant lymphoma, and pancreas cancer with and without CLD patients were analyzed.

LORA: late-onset **rheumatoid arthritis**, CLD: chronic lung disease

Supplementary Table S3 Factors associated with SAEs of special interest

|  | Adjusted HR (95% CI) | *p*-value |
| --- | --- | --- |
| Age, years | 1.03 (0.99–1.08) | 0.145 |
| Sex, female | 1.02 (0.55–1.89) | 0.947 |
| SDAI at week 0 | 1.01 (0.99–1.02) | 0.36 |
| HAQ-DI at week 0 | 1.10 (0.80–1.51) | 0.547 |
| GC use | 1.41 (0.85–2.35) | 0.187 |
| Ccr <60 ml/min at baseline | 1.40 (0.84–2.33) | 0.199 |
| Osteoporosis at baseline | 1.25 (0.71–2.21) | 0.438 |
| History of infections requiring hospitalization | 1.20 (0.57–2.54) | 0.625 |
| History of smoking | 1.36 (0.77–2.40) | 0.295 |
| History of malignancy | 2.37 (1.28–4.38) | 0.006* |
| CLD at baseline | 2.53 (1.60–4.00) | <0.001* |

Multivariate Cox proportional hazards analysis was performed with age, SDAI, HAQ-DI, GC use, history of smoking and comorbidities (Ccr <60 ml/min, osteoporosis, history of infections requiring hospitalization, history of malignancy, history of Chronic lung disease) at baseline as covariates.

CLD: chronic lung disease, SDAI: Simplified Disease Activity Index, HAQ-DI: Health Assessment Questionnaire Disability Index, GC: glucocorticoid, Ccr; creatinine clearance,

^*^Statistically significant with p < 0.05.
